# Understanding Conditionals in the East: A Replication Study of Politzer et al. (2010) With Easterners

**DOI:** 10.3389/fpsyg.2018.00505

**Published:** 2018-04-12

**Authors:** Hiroko Nakamura, Jing Shao, Jean Baratgin, David E. Over, Tatsuji Takahashi, Hiroshi Yama

**Affiliations:** ^1^Department of Human Informatics, Aichi Shukutoku University, Nagakute, Japan; ^2^CHArt (PARIS), Université Paris 8 and EPHE, Saint-Denis, France; ^3^Université de Haute-Alsace, Mulhouse, France; ^4^Institut Jean Nicod, École Normale Supérieure, Paris, France; ^5^Department of Psychology, Durham University, Durham, United Kingdom; ^6^School of Science and Engineering, Tokyo Denki University, Tokyo, Japan; ^7^Faculty of Literature and Human Sciences, Osaka City University, Osaka, Japan

**Keywords:** new paradigm psychology of reasoning, indicative conditionals, bets on conditionals, de Finetti table, cultural differences

## Abstract

The new probabilistic approaches to the natural language conditional imply that there is a parallel relation between indicative conditionals (ICs) “*if s then b*” and conditional bets (CBs) “*I bet $1 that if s then b*” in two aspects. First, the probability of an IC and the probability of winning a CB are both the conditional probability, *P*(*s|b*). Second, both an IC and a CB have a third value “void” (neither true nor false, neither wins nor loses) when the antecedent is false (*¬s*). These aspects of the parallel relation have been found in Western participants. In the present study, we investigated whether this parallel is also present in Eastern participants. We replicated the study of Politzer et al. (2010) with Chinese and Japanese participants and made two predictions. First, Eastern participants will tend to engage in more holistic cognition and take all possible cases, including *¬s*, into account when they judge the probability of conditional: Easterners may assess the probability of antecedent *s* out of all possible cases, *P*(*s*), and then may focus on consequent *b* out of *s*, *P*(*b|s*). Consequently, Easterners may judge the probability of the conditional, and of winning the bet, to be *P*(*s*) *^∗^ P*(*b|s*) = *P*(*s* & *b*), and false/losing the bet as *P*(*s*) *^∗^ P*(*¬b|s*) = *P*(*s* & *¬b*). Second, Eastern participants will tend to be strongly affected by context, and they may not show parallel relationships between ICs and CBs. The results indicate no cultural differences in judging the false antecedent cases: Eastern participants judged false antecedent cases as not making the IC true nor false and as not being winning or losing outcomes. However, there were cultural differences when asked about the probability of a conditional. Consistent with our hypothesis, Eastern participants had a greater tendency to take all possible cases into account, especially in CBs. We discuss whether these results can be explained by a hypothesized tendency for Eastern people to think in more holistic and context-dependent terms than Western people.

## Introduction

The new Bayesian and probabilistic accounts of the natural language indicative conditional (IC) have become increasingly influential in the psychology of reasoning. The older *binary account* of reasoning was based on binary logic and classified propositions as simply true or false. Such a classification, however, is too simple for most ordinary and scientific reasoning, which takes place in a context of uncertainty, where many propositions are neither certainly true nor certainly false. In contrast, the *new paradigm* in the psychology of reasoning recognizes that people have degrees of belief in propositions, which are technically subjective probabilities and that this affects even the fundamental logic of their reasoning (Oaksford and Chater, 2007, 2010; Over, 2009; Politzer et al., 2010; Baratgin et al., 2013; Baratgin and Politzer, 2016; Over and Baratgin, 2017).

In this new approach, the probability of an IC of natural language *P*(*if p then q*) is equal to the conditional probability of *q given p*, *P*(*q|p*), and not to the probability of the formal conditional of binary logic, which is logically equivalent to *not-p or q*. The statement that *P*(*if p then q*) is not *P*(*not-p or q*) but rather *P*(*q|p*), *P*(*if p then q*) = *P*(*q|p*), is often called *the Equation*, because it has such far-reaching implications for a Bayesian account of conditional reasoning. It is based on logical and philosophical studies of conditionals (Ramsey, 1929/1990; Edgington, 1995; Adams, 1998), which suggest that people assess *P*(*if p then q*) by hypothetically supposing *p* and then judging the probability of *q* given under this supposition of *p*, a process that is called the *Ramsey test*. If the Equation holds, then a Bayesian account of conditional reasoning will follow, with Bayesian probability theory, and not binary logic, as the new normative standard, a new paradigm, for conditional reasoning (Evans and Over, 2004; Oaksford and Chater, 2007; Pfeifer and Kleiter, 2010; Baratgin and Politzer, 2016). Also in this new approach, a conditional can have a third value, “neither true nor false (void),” in addition to truth or falsity, when its antecedent is false. We will follow recent practice and call the resulting truth table a *de Finetti* table, after de Finetti (1936/1995) who first proposed it (see Baratgin et al., 2013, 2014). The Equation has been very strongly supported for people’s probability judgments about natural language conditionals *if p then q*, at least when *p* and *q* are not independent of each other (Evans et al., 2003; Oberauer and Wilhelm, 2003; Over et al., 2007; Douven and Verbrugge, 2010; 2013; Fugard et al., 2011; Singmann et al., 2014; Baratgin et al., 2017). For an even longer time, there has been confirmation of the *de Finetti table*, traditionally called the “defective” truth table, as descriptive of people’s judgments about ICs (Over and Baratgin, 2017). Note that Jeffrey (1991) proposed a many-valued extension of the *de Finetti* table, the *Jeffrey table*. This proposal is an important generalization that is arguably found in de Finetti himself, and it should be investigated in future research (Over and Baratgin, 2017).

The new approach implies in turn that there should be a close parallel relation between the assertion of an IC and a conditional bet (CB) as a speech act (de Finetti, 1937/1964; Politzer et al., 2010; Baratgin et al., 2013). Making a connection between betting, including CBs, and subjective probability theory goes right back to the origin of contemporary subjective probability theory, together with the use of betting quotients to represent degrees of belief and the Dutch book arguments to justify the axioms of probability theory (Ramsey, 1926/1990; de Finetti, 1937/1964, 1974). The parallel relation between an IC and a CB can easily be illustrated with an example:

(1) If it rains in Kobe tomorrow, the baseball match will be called off.
(1) I bet that, if it rains in Kobe tomorrow, the baseball match will be called off.

Suppose a Kobe baseball fan asserts (1) and then goes on to (2) in an argument with someone who denies (1). Such speech acts often occur together, or interchangeably, in discussions and debates about singular matters of fact (see Cruz and Oberauer, 2014, on general conditionals). In more detail, the parallel relation is as follows. The fan’s assertion (1) is true, and she wins her bet (2), when it rains in Kobe, and the match is called off. The fan’s assertion (1) is false, and she loses her bet (2), when it rains in Kobe, and the match is not called off. The fan’s indicative assertion is not shown to be true or false, and she neither wins nor loses her bet when it does not rain in Kobe. In this case, the IC and CB are both *void*. No actual fact makes the indicative assertion true or false, and the CB is called off, with no money changing hands. In any case, the probability that (1) holds is the conditional probability that the baseball match will be called off given that it rains in Kobe tomorrow. The probability that the fan will win her CB (2) is also this conditional probability, which is indeed the fair betting quotient for the bet. The conditional probability can be determined by means of the *Ramsey test*, which is to suppose that it will rain tomorrow in Kobe and then to infer, under this supposition, a degree of belief that the baseball match will be called off (Ramsey, 1929/1990; Edgington, 1995).

The new paradigm implies that there should be this parallel relation between conditional assertions, like (1), and CBs, like (2), and Politzer et al. (2010) have investigated whether people’s judgments are broadly in line with it. They used an IC and CB about a randomly selected chip from a given frequency distribution of chips that were square or circular and black or white:

(1) If the chip is square (*s*), then it is black (*b*).
(1) I bet you 1 Euro that if the chip is square, then it is black.

More than 60% of their participants did respond that the probability of the IC (3), of the form *if s then b*, and the probability of winning the CB (4), of the form *I bet that if s then b*, were both equal to the conditional probability, *P*(*b|s*). More than 50% of the participants also judged that the false antecedent outcomes, *¬s* & *b* and *¬s* & *¬b*, made the IC neither true nor false and resulted in a CB being neither won nor lost. There was no evidence that the participants interpreted the IC as the *material conditional* of elementary formal logic, which is logically equivalent to *not-s or b*, and no evidence that they interpreted the CB as a bet on *not-s or b*.

There were, however, some responses that are hard to classify. About 15% of the participants judged that the probabilities of the IC and the CB were equal to conjunctive probability *P*(*s* & *b*). And in false antecedent *¬s* & *b* and *¬s* & ¬*b* cases, 28% of the participants judged that the IC is false, and 10% of participants judged that the CB is lost. These *conjunctive responses* are clearly inconsistent with any reasonable normative theory of the conditional, but have been found in other experiments on ICs like (3), which are about randomly selected objects from artificial frequency distributions (e.g., Evans et al., 2003). Conjunctive responses are not found at all when probability judgments are made about everyday conditionals that are not about such frequency distributions, e.g., “If global warming continues, then Hamburg will be flooded” (Over et al., 2007; Singmann et al., 2014). Even so, the conjunctive response must obviously be explained for the contents and contexts in which it occurs.

Politzer et al. (2010) suggested that some people might respond with the conjunctive probability, *P*(*s* & *b*), because it takes fewer mental steps to process it than the conditional probability, *P*(*b|s*), when an unfamiliar frequency distribution is being referred to. To respond with *P*(*s* & *b*), one only has to consider the total number of *s* & b chips out of all the chips, but to derive *P*(*b|s*), one must first restrict one’s attention to the *s* chips alone and then consider the proportion of these chips are *s* & *b* chips. The former process is easier than the latter. This explanation is supported by the results of other studies of ICs. Conjunctive responses tend to decline and become replaced by conditional probability responses as participants become more practiced at making these probability judgments about initially unfamiliar frequency distributions (Fugard et al., 2011). Studies of individual differences show that conditional probability responders are of higher cognitive ability than conjunctive probability responders (Evans et al., 2007; Oberauer et al., 2007).

These results on conjunctive responders and ICs may not transfer to CBs. Betting is a social activity that is about winning or losing money or some other utility. It may be that there are special reasons, individual or cultural, why some people give the conjunctive response to questions about CBs, and yet there are only two studies of them (Politzer et al., 2010; Baratgin et al., 2013). These studies are also restricted to participants in Western Europe. There is certainly a need for further studies of both ICs and CBs in non-Western cultures, as there are claims about cultural differences in cognition in the literature that have some support.

Nisbett and his collaborators (Nisbett et al., 2001; Nisbett and Masuda, 2003; Nisbett and Miyamoto, 2005) have argued, on the basis of their results, that Westerners are more engaged in analytic and context-independent cognition, and Easterners are more concerned with holistic and context-dependent cognition and also what is termed “naïve dialecticism.” For example, studies of scene cognition (Masuda and Nisbett, 2001) found that American participants separated a salient object from its background and focused on it, while Japanese participants combined the salient object and the background and paid more attention to the scene as a whole.

Norenzayan et al. (2002) studied cultural differences in syllogistic reasoning. In their study, East Asian (Korean) participants showed stronger belief bias than European and American participants, although there were no cultural differences in logical accuracy for abstract arguments. They concluded that there are not cultural differences in logical reasoning ability, but rather cultural differences in context dependency.

There are also claimed differences in interpreting contradictions and compliance with binary logic. It has been argued that Easterners tend to be inclined to “naïve dialecticism” (Peng and Nisbett, 1999; Spencer-Rodgers et al., 2009, 2010). In this view, the universe is in a state of flux and alternates between opposites (e.g., good becomes bad, but then bad becomes good). Naïve dialectical thinkers are tolerant of apparent contradictions and have a belief that the “truth” is often somewhere in the middle. In contrast, Westerners are supposedly guided by the law of non-contradiction, and a belief that all propositions must be either true or false, and are more inclined to follow binary logic to evaluate propositions. “Dialecticism,” so defined, appears to some extent in line with the new paradigm in the psychology of reasoning and its use of probability theory, and not truth functional logic, as the normative standard for conditional reasoning. Easterners’ conditional reasoning might then conform more to a probabilistic account of conditionals, with a greater tendency to judge the probability of a conditional as the conditional probability and to evaluate false antecedent cases as void. On the other hand, “void” expresses a tolerance for what is not factually true or false, and it might be that Easterners take a view of void outcomes different from that of Westerners. Hence, Easterners’ interpretation of the probability of a conditional might differ from Westerners’ interpretation. This possibility has not yet been investigated in a cross-cultural study. Possible cultural differences may or may not affect the understanding and evaluation of ICs, and CBs, but if they do, a full theory of conditional reasoning would have to account for them.

## Overview of the Experiments

Our study of conditionals recruited Chinese and Japanese participants as Easterners, and replicated the same experimental paradigm as in Politzer et al. (2010), who had French participants. The participants were presented with an IC or a CB, and asked to judge the probability that the IC was true, or false, and the probability of winning, or losing, the CB. They were also asked to evaluate the truth value of the IC, *if s then b*, in false antecedent *¬s* cases, and to judge whether the CB was won, lost, or called off in these cases. Norenzayan et al. (2002) showed that there were no cultural differences in an abstract syllogistic reasoning. Therefore, it is possible that there are no cultural differences in the understanding abstract conditionals, and Easterners may understand a conditional *if s then b* as suppositional as *b given s*.

However, in light of the literature referred to the above, there are two hypotheses to consider about possible differences in conditional reasoning between Easterners and Westerners. The first hypothesis is that Easterners tend to engage more in holistic cognition about the conditional, viewing all the logical possibilities, including the false antecedent *¬s* cases, as relevant to its evaluation. Because Easterners tend to engage in naïve dialecticism and have greater tolerance of inconsistency (Spencer-Rodgers et al., 2009), they may evaluate *¬s* cases as relevant when they judge the probability of a conditional, or of winning/losing the bet, even though they understand a conditional as suppositional as *b given s* and evaluate *¬s* cases as void (neither true nor false, neither wins nor loses). We predict that Easterners might make *holistic* responses. When Easterners think about the probability of conditional *if s then b*, they might pick *s* chips out of all chips, *P*(*s*), before they focus on *b* chips out of *s* chips, *P*(*b|s*). Therefore, Easterners may judge that the probability of the conditional being true, and the probability of a win, is equal to *P*(*s*) *^∗^ P*(*b|s*) = *P*(*s* & *b*), and the probability of conditional being false, and of losing, is *P*(*s*) *^∗^ P*(*¬b|s*) = *P*(*s* & *¬b*). Note that the holistic way of determining *P*(*s* & *b*), which takes a subset of a subset [specifically, *b|s* out of *s*, or equivalently, *P*(*s*) *^∗^ P*(*b|s*)], is different from the conjunctive response *P*(*s* & *b*), which just focuses on the number of *s* & *b* chips out of the total number of all chips.

The second hypothesis, which might hold along with the first or on its own, is that Easterners are more strongly affected by contextual information (Norenzayan et al., 2002) and they may evaluate bets, and specifically CBs, differently from Westerners. As we have already pointed out, betting is a social activity, which may vary between cultures. With supposedly more holistic thought, Easterners might again consider *¬s* cases as relevant to the evaluation of the CB, a bet on *if s then b*. They might consider a *¬s* case as a losing outcome, because no money is won, and not as a neither winning nor losing, void outcome. There would then be relatively more conjunctive responses among Easterners for CBs.

## Materials and Methods

**FIGURE 1:**
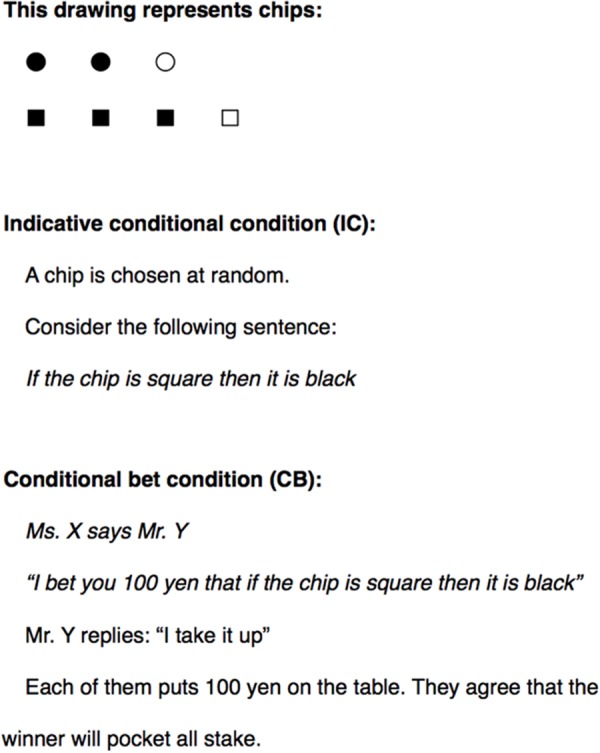
The display of the chips and the conditional sentences in the indicative conditional and the conditional bet conditions in the experiment.

We investigated the above two hypotheses using the approach of Politzer et al. (2010) with Chinese and Japanese participants. The experimental procedure was identical with that of Politzer et al. (2010). The participants were presented with an IC *If the chip is square, then it is black*, and a CB *I bet you ¥100 that if the chip is square, then it is black* about a randomly selected chip from a distribution of square or circular and black or white chips (**Figure 1**). Seven chips were shown to them: three square and black chips, one square and white chip, two circular and black chips, and one circular and white chip. The relevant conditional probabilities were thus *P*(*b*|*s*) = 3/4 and *P*(*¬b*|*s*) = 1/4. The probability of the conjunction was *P*(*s* & *b*) = 3/7 and *P*(*¬*(*s* & *b*)) = 4/7, and the probability of the material conditional was *P*(*¬s* or *b*) = 6/7, with *P*(*s* & *¬b*) = 1/7. There is also the *expected value* (EV) of the CB to keep in mind, which is given by EV = *P*(*s* & *b*)(*100*) + *P*(*s* & *¬b*)(*-100*) + *P*(*¬s*)(*0*) = (3/7)(100) + (1/7)(-100) = 29. Clearly, this is not a fair bet, which would otherwise have an EV of 0, and which would come from odds of 3 to 1 given by the fair betting quotient for this CB, *P*(*b*|*s*) = 3/4. But many of the bets that ordinary people enter into, as in a casino, are not fair in this strict sense.

### Participants^1^

Chinese participants were 175 undergraduate students of Zhejiang University of Media and Communications (38 male, 137 female, *M*_age_ = 19.0, *SD*_age_ = 1.4). Japanese participants were 197 undergraduate students of Osaka City University and Hokkaido University (85 male, 110 female, 2 unknown, *M*_age_ = 20.1, *SD*_age_ = 3.2). The experiment was conducted anonymously and we obtained written informed consent from all participants. This study was carried out in accordance with the recommendations of “Ethical Principles of Psychologists and Code of Conduct, The American Psychological Association.”

### Materials

We translated the experimental material used in Politzer et al.’s (2010) study (Groups 1–6) into Chinese and Japanese. There were six questions about chips in the two colors (white or black) and two shapes (circular or square), along with conditional statements about the chips. Questions 1–4 were about three circular chips (two black and one white) and four square chips (three black and one white). Question 5 and 6 were about four circular chips (one black and four white) and three square chips (one black and two white).

### Design

Participants were randomly allocated to four groups. Groups 1 and 2 received the IC, *If the chip is square, then it is black*, and they answered six questions about IC. Groups 3–6 received a CB, in the form Ms. X says to Mr. Y, *I bet you ¥100 that if the chip is square, then it is black*, and answered six questions about CB.

### Questions 1 and 2 (Probability Judgment)

Groups 1 and 2 were asked to judge the probability that the IC is true or false. Question 1 was: *what are the chances that the sentence is true?* Question 2 was: *what are the chances that the sentence is false?* Groups 3–6 were asked for the probability that Ms. X would win or lose her bet. Question 1 for them was: *what are the chances that Ms. X will win her bet?* Question 2 for them was: *what are the chances that Ms. X will lose her bet?* Participants were asked to write down the mathematical formula for calculating the probability.

### Questions 3 and 4

#### Evaluation of ¬*s* Cases

Groups 1–4 were presented with the false antecedent outcomes. Question 3 was about *¬s* & *b* outcome*: suppose that the chosen chip is round and black*. Question 4 was about *¬s* & *¬b* outcome*: suppose that the chosen chip is round and white*. Groups 1 and 2 were asked whether the conditional was true or false, and Groups 3 and 4 were asked whether the CB was won or lost in false antecedent cases. Group 1 had a two-option question: *do you think that the sentence is true, or false?* Group 2 had a three-option question: *do you think that the sentence is true, false, or neither true nor false (void)?* Group 3 had a two-option question about whether Ms. X won or lost her bet. Group 4 had a three-option question about whether Ms. X won, lost, or had her bet called off (made void). For example, Group 4 had the following for Question 4. *Suppose that the drawn chip is round and black. Do you think that Ms. X wins her bet, loses her bet, or nobody wins (the bet is called off)?*

#### Probability of *b* and *¬b* Cases

Groups 5 and 6 were also given the CB, and they were asked to make a judgment under the supposition that the chosen chip was a black, a *b* outcome, or that it was white, a *¬b* outcome. Group 5 was asked to judge the probability of wining the bet given that the chip is black *P*(*win|b*) in Question 3, and then they were asked the probability of losing the bet given that the chip is black *P*(*lose|b*) in Question 4. For example, “*Suppose that the chip is black. What are the chances that Ms. X wins her bet?*” Group 6 was asked to judge the probability of wining the bet given that the chip is white, *P*(*win|¬b*), in Question 3, and then they were asked the probability of losing the bet given that the chip is white, *P*(*lose|¬b*) in Question 4. For example, “*Suppose the chip is white. What are the chances that Ms. X wins her bet?*”

### Questions 5 and 6

Questions 5 and 6 were given to all groups and in a counterbalanced order. To avoid a possible effect of training, these questions were about a different set of chips: three square chips, one black and two white, and four circular chips, one black and three white. These questions were about the probability of the conjunction, *P*(*s* & *b*) and about the conditional probability, *P*(*b|s*). The aim of these questions was to test whether the participants could distinguish a request to evaluate the probability of conjunction from a request to evaluate the conditional probability.

## Results

We include the French data from Politzer et al. (2010) in **Tables 1**–**3**, in order to test the differences between Easterners and Westerners.

### Questions 1 and 2 (Probability Judgment)

**Table 1:** Percentage of responses of French, Chinese, and Japanese participants in probability judgment task (Questions 1 and 2).

Response category		Indicative conditional			Conditional bet		
			
		French	Chinese	Japanese	French	Chinese	Japanese
Conditional probability	(3/4 for true/win, 1/4 for false/lose)	69	51	62	61	37	36
Conjunctive probability	(3/7 for true/win, 4/7 for false/lose)	14	7	8	16	9	18
Material implication	(6/7 for true/win, 1/7 for false/lose)	2	0	3	2	1	4
Holistic		0	15	16	2	25	30
Holistic 1	(3/7 for true/win, 1/7for false/lose)	(0)	(7)	(14)	(1)	(11)	(15)
Holistic 2	(4/7 ^∗^ 3/4 = 3/7 for true/win, 1 - 3/7 = 4/7 for false/lose)	(0)	(8)	(2)	(1)	(14)	(15)
Other		15	27	11	19	28	12


**Table 2:** Percentage of responses of French, Chinese, and Japanese participants in *¬s* evaluation task (Questions 3 and 4 in Groups 1–4).

	Indicative conditional			Conditional bet		
		
	French	Chinese	Japanese	French	Chinese	Japanese
Groups 1 and 3: two-choice option						
True/win for both questions	37	24	30	13	32	19
False/lose for both questions	47	48	43	40	22	47
True/win for one false/lose for the other	3	24	24	20	32	19
Neither true nor false/void to both questions^∗^	13	0	0	27	7	12
Mixed	0	4	3	0	7	3
Groups 2 and 4: three-choice option						
True/win for both questions	7	7	8	7	3	6
False/lose for both questions	28	13	19	10	0	13
True/win for one false/lose for the other	3	4	3	3	14	6
Neither true nor false/void to both questions	52	63	54	79	80	75
Mixed	10	13	16	0	3	0


*^∗^This is spontaneous expression of the third option.*

**Table 3:** Percentage of responses of French, Chinese, and Japanese participants in probability judgment about *b* or *¬b* outcomes (Questions 3 and 4 in Groups 5 and 6).

Q3. Suppose the chip is black. What are the chances that Mary wins her bet?	French	Chinese	Japanese	Q4. Suppose the chip is black. What are the chances that Mary loses her bet?	French	Chinese	Japanese
**Group 5**							
1 (or 100%)	0	17	27	0 (or 0%)	7	13	53
3/5 = *P*(*s|b*)	55	60	47	2/5 = *P*(¬*s|b*)	59	64	23
3/4 = *P*(*b|s*)	18	3	3	3/4 = *P*(*b|s*)	7	0	0
5/7 = *P*(*b*)	10	0	0	1/4 = *P*(¬*b|s)*	7	3	0
Other	17	20	23	Other	20	20	24
**Group 6**							
0 (or 0%)	47	55	59	1 (or 100%)	27	42	31
1/2 = *P*(*¬s|¬b*)	17	21	28	1/2 = *P*(*s|¬b*)	20	28	59
2/7 = *P*(¬*b*)	10	0	0	0 (or 0%)	13	3	3
1/4 = *P*(¬*b|s*)	10	0	0	3/4 = *P*(*b|s*)	10	0	0
Other	16	24	13	1/4 = *P*(¬*b|s*)	10	3	0
				Other	20	24	7


**Table 1** shows the percentage of answers to the probability judgment task in Groups 1–6. We included in this table an entry for *holistic 1* and *holistic 2* responses. Holistic 1 response was defined to be the ones that focus on the probability of the true or winning case *P*(*s*) *^∗^ P*(*b|s*) = *P*(*s* & b) = 3/7, and of the false or losing case *P*(*s*) *^∗^ P*(*¬b|s*) = *P*(*s* & *¬b*) = 1/7. Some participants calculated that the probability of a conditional is true/win with the following formula, *P*(*s*) *^∗^ P*(*b|s*) = 4/7 ^∗^ 3/4 = 3/7, and calculated that the probability of a conditional is false/lose as 1 – 3/7 = 4/7. Because this calculation is of the same nature of holistic 1 response, different from conjunction, we counted this response as *holistic 2* response, and not as conjunctive probability response.

In this and subsequent analysis, we used 2 ^∗^ 3 Fisher’s exact test to compare response frequencies because there were a small number of response in some categories (i.e., the number of holistic responses in French was 0 in IC condition). For example, to compare frequency of conditional probability response in IC, we conducted a 2 ^∗^ 3 Fisher’s exact test; 2 (response category: conditional probability response, non-conditional probability response) ^∗^ 3 (nationality: French, Chinese, Japanese). When there were differences in response frequency, we conducted a 2 ^∗^ 2 Fisher’s exact test for pairwise comparison three times that were 2 (response category) ^∗^ 2 (nationality: French, Chinese), 2 (response category) ^∗^ 2 (nationality: French, Japanese), and 2 (response category) ^∗^ 2 (nationality: Chinese, Japanese). In pairwise comparison, we apply Bonferroni correction for multiple test and *p*-value was set to less than 0.0167 (0.05/3) to be significant at the *p* < 0.05 level.

The frequency of conditional probability response did not differ between the three countries in IC (*p* > 0.05), but there were differences in CB (*p* < 0.001). In CB, the frequency of conditional probability response was greater in French than in Chinese (*p* < 0.001) and in Japanese (*p* < 0.001), but there was no difference between Chinese and Japanese (*p* > 0.05). There were no differences between the three countries in the frequency of conjunctive probability response and the frequency of material implication response both in IC and in CB (*p* > 0.05). As we predicted, we observed significant differences in the frequency of the holistic response (holistic 1 and holistic 2) both in IC (*p* < 0.001) and in CB (*p* < 0.001). The frequency of the holistic response was greater in Chinese and in Japanese than in French, but there were no differences between Chinese and Japanese both in IC (French and Chinese *p* < 0.05, French and Japanese *p* < 0.01, Chinese and Japanese *p* > 0.05) and in CB (French and Chinese *p* < 0.001, French and Japanese *p* < 0.001, Chinese and Japanese *p* > 0.05). In sum, there were cultural differences in response frequency in judging the probability of conditionals. Whereas the conditional probability response was given more often by the French participants than by the Eastern participants (in both conditions, but significantly so only in CB), the holistic response was given more often by the Eastern participants than by the French participants (significant both in IC and in CB).

### Questions 3 and 4 in Groups 1–4 (Evaluation of ¬s Cases)

**Table 2** shows the distribution of the answers for evaluating *¬s* outcomes in Groups 1 and 3 (two-choice questions) and Groups 2 and 4 (three-choice questions). Note that Groups 1 and 3 presented two-choice option questions such as “*Do you think that the sentence is true or false?*” some participants did not select options but wrote down third options such as “*neither true nor false*” or “*neither wins nor loses*” and we categorized these responses as “void.” Therefore, we included “void” choices as well as true/win and false/lose responses for analyzing response frequencies.

As for the response frequency for two-choice questions (Groups 1 and 3) in IC, there were no differences between the three countries in true for both, false for both, and mixed (*p* > 0.05). There was a significant difference in one for true and one for false response (*p* < 0.05), while pairwise comparison showed no statistically significant difference. We also observed a significant difference in the frequency of “void” for both response (*p* < 0.05), but no statistically significant difference in pairwise comparison. Groups 1 and 3 in CB showed no difference between the three countries in all response categories (win for both, lose for both, win for one and lose for the other, “void” for both, *p* > 0.05).

Three-choice questions (Groups 2 and 4) showed no significant differences between the three countries in all response categories both in IC (true for both, false for both, true for one and false for the other, and void for both, *p* > 0.05) and in CB (win for both, lose for both, true for one and false for the other, and void for both, *p* > 0.05). In sum, there was no cultural difference in evaluation of truth value of false antecedent outcomes, and the modal response was void in three-choice option questions.

### Questions 3 and 4 in Groups 5 and 6 (About b and ¬b Cases)

**Table 3** shows the distribution of the answers for Questions 3 and 4 in Groups 5 and 6. Group 5 was asked to suppose the chip is black (*b*) and to estimate the probability of winning (Q3) or losing (Q4) the bet. Group 6 was asked to suppose the chip is white (*¬b*) and to estimate the probability of winning (Q3) or losing (Q4) the bet.

In Question 3 in Group 5 (probability of winning in black chip), there was a difference in frequency of response “1 (100%)” (*p* < 0.01) and the frequency of response “1 (100%)” was greater in Japanese than in French (*p* < 0.01), but there was no difference between French and Chinese and Chinese and Japanese (*p* > 0.05). In frequency of responses “3/5” and “3/4,” there was no difference between the three countries (*p* > 0.05). There was a significant difference in frequency of response “5/7” (*p* < 0.05), but pairwise comparison showed no significant differences.

In Question 4 in Group 5 (probability of losing in black chip), there were differences in frequency of response “0 (0%)” (*p* < 0.001) frequency of response “0 (0%)” was greater in Japanese than in French (*p* < 0.001) and in Chinese (*p* < 0.001), but there were no differences between French and Chinese (*p* > 0.05). We also observed differences in the frequency of response “2/5” (*p* < 0.01): the frequency of response “2/5” was greater in French than in Japanese (*p* < 0.01) and greater in Chinese than in Japanese (*p* < 0.01), but there were no differences between French and Chinese (*p* > 0.05). There were no significant differences in frequency of response “3/4” and response “1/4” (*p* > 0.05). Together with these results, there were cultural differences in judging the probability of winning or losing in *b* outcomes: Japanese participants more frequently judged that the probability of winning in *b* was 100% and the probability of losing in *b* was 0%, but less frequently judged that the probability of losing in *b* was 2/5.

Question 3 in Group 6 (probability of winning in white chip) showed no difference between the three countries in all response categories (*p* > 0.05 for responses “0,” “1/2,” “2/7,” and “1/4”).

In Question 4 in Group 6 (probability of losing in white chip), there was a difference in frequency of response “1/2” (*p* < 0.001), and pairwise comparison indicated that the frequency of response “1/2” was greater in Japanese than in French (*p* < 0.001) and in Chinese (*p* < 0.05), but there was no difference between French and Chinese (*p* > 0.05). There were no differences between the three countries in the other response categories (*p* > 0.05 for responses “1,” “0,” “3/4,” and “1/4”).

## Discussion

The purpose of the present study was to test Bayesian accounts of the natural language conditional with Easterners by replicating the study of Politzer et al. (2010). These accounts imply that there is a parallel relation between ICs and CBs: both IC and CB are related to the conditional probability, *P*(*b|s*), and both have a *de Finetti* table where the false antecedent cases, *¬s*, are void (neither true nor false/neither wins nor loses). This parallel relationship has been found in the judgments of Western people about IC and CB (Politzer et al., 2010; Baratgin et al., 2013).

We considered the hypothesis that Easterners are more strongly engaged in holistic cognition and pay more attention to a context as a whole, with the result that both Chinese and Japanese participants would have a greater tendency, compared to French participants, to view *¬s* cases as relevant, rather than irrelevant, to the evaluation of conditionals. And our results showed cultural differences, where the response in Easterners did not equal to the normative standard of the new paradigm position that *P*(*if s then b*) = *P*(*b|s*). We argued that the basis of this response was not merely a simple mistake, but cultural differences in cognition might cause the holistic response in Easterners.

In the results, the modal response in all the three countries of a probability judgment task was the conditional probability response and was “void” for evaluating *¬s* cases. However, there were cultural differences in response frequency in judging the probability of conditionals. Consistent with our hypothesis, Eastern participants made more holistic responses: they counted *s* chips out of *all* these chips for first *P*(*s*), and then they focused on the true/winning chips *b* out of *s* chips *P*(*s*) ^∗^ *P*(*b|s*) = *P*(*s* & *b*). When Easterners judged the probability of false/lose, they also count *s* chips out of *all* chips *P*(*s*), and then focused on the false/losing chips ¬*b* out of *s* chips *P*(*s*) *^∗^ P*(*¬b|s*) = *P*(*s* & *¬b*), or some subtracted chance of true/win out of all cases, 1 -*P*(*s* & *b*). Furthermore, Eastern participants less frequently made conditional probability responses than French participants in CB.

Note that the numerical value of a holistic response is the same as that of conjunctive response *P*(*s* & *b*), but the process is different. The conjunctive response just counts the number of *s* & *b* chips out of all chips, while a holistic response needs to assess the occurrence of *s*. Although some French participants made a conjunctive response, only 2% of them wrote down a calculation consistent with a holistic response. In addition, with an eye tracking methodology, Baratgin et al. (2017) for the IC condition and de Gans (2017, Unpublished) for the bet condition found that French participants give the simple immediate responses of the ratios for the *P*(*b|s*) and *P*(*s* & *b*) answers. In de Gans (2017, Unpublished), all participants who gave the conjunction answer simply used the ratio (black squares)/(total number of chips). Therefore, holistic response, *P*(*if s then b*) = *P*(*s*) *^∗^ P*(*b|s*), is so prominently found in the Asian participants.

Our results indicate that Easterners had a greater tendency to take *¬s* cases into account when they evaluated the conditionals, even though they classified *¬s* cases as void (neither true nor false/neither wins nor loses). In addition, in a holistic response, the chance of true/win (3/7) and false/lose (1/7) did not sum up to 100%. Therefore, Easterners may have more tolerance for contradictions, or alternatively Easterners’ interpretation of void value may be different from that of Westerners. It is possible that Eastern participants were cognitive misers and did not consider contradictions. However, there were no cultural differences in the frequency of the conjunctive probability response, which might be due to mental shortcutting (Evans et al., 2007). It is also possible that Eastern participants are sensitive to contextual information, and they might calculate an EV in bet contexts. Our CB material had a positive EV with a value of 29 yen, and therefore participants might think of “*lost*” of this positive EV in a *¬s* case, in which the bet is called off. However, in the evaluation of false antecedent, *¬s* cases, we did not observe any cultural difference.

Therefore, there are cultural differences in judging the probability of conditionals, and these cultural differences might not relate to mental shortcutting or the EV in bet contexts. There might rather be differences in analytic–holistic cognition (Nisbett et al., 2001), naïve dialecticism (Spencer-Rodgers et al., 2009), and in a context-dependent tendency (Norenzayan et al., 2002). The tendency in Eastern participants for holistic cognition and naïve dialecticism might give then a greater tendency to take the whole set of cases into account and Easterners might consider that *¬s* is void case (neither true nor false/neither wins nor loses) but not irrelevant to the conditionals.

Furthermore, Eastern participants tended to be affected by context, and the bet context might increase the availability of *¬s* cases. Because “*bet is called off*” is more familiar and more available than “*neither true nor false*,” Easterners might consider false antecedent *¬s* cases as relevant to the bet as well as winning and losing cases. Consequently, Easterners made fewer conditional probability responses, which did not take *¬s* cases into account, in the bet context.

In probability judgments about *b* and *¬b* cases, there was no difference between Chinese and French participants, and the results were similar to Politzer et al. (2010). For these questions, the modal response of winning, given *b*, was *P*(*s|b*), and of losing, given *b*, was *P*(*¬s|b*). The modal response of winning, given *¬b*, was 0, and of losing, given *¬b*, was 1. Politzer et al. (2010) explained these results as follows: participants tend to polarize toward winning outcomes, and they consider void cases as failure to win the bet; consequently, French participants counted *¬s* & *b* and *¬s* & *¬b* cases as losing outcomes, as well as the most basic losing outcome of *s* & *¬b*. Although both French and Chinese participants showed a similar response pattern in these cases, it is possible that Chinese participants calculated differently from French participants. Compared with French participants, Chinese participants made more holistic two responses, *P*(*losing*) = 1 – *P*(*winning*), in the probability judgment task; therefore, Chinese might have calculated the probability of winning first, and then calculated the probability of losing by subtraction, *P*(*losing*) = 1 – *P*(*winning*), instead of counting *¬s* & *b* and *¬s* & *¬b* as losing outcomes.

In the probability judgments about *b* and *¬b* cases, we observed differences between Japanese and French as well as Japanese and Chinese: Japanese participants more frequently answered that the probability of winning, given *b*, was 1, the probability of losing, given *b*, was 0, and losing given ¬b, was 1/2. Because Japanese participants tend to engage in holistic cognition, they might have a greater tendency to consider all possible *b* outcomes, including the “called off,” *¬s*, cases. Similarly, Japanese participants might have a greater tendency to judge *s* & *¬b* outcomes as losing and *¬s* & *¬b* as “called off” outcomes in Group 6 and to judge the probability of losing, given ¬*b*, as 1/2. Although there are few cross-cultural psychological studies comparing differences between Chinese and Japanese participants, the meta-analytic study of Oyserman et al. (2002) implied that the Chinese are less individualistic and more collectivistic than the Japanese. It is then possible that Chinese participants might focus on all the possible outcomes and subtract the winning cases from these. Japanese participants might also focus on the all the cases, but since they may be relatively more individualistic than the Chinese, they might tend more to divide all the cases into the components of winning, losing, and “called off.”

There were further differences in response frequencies of the “void” response in the two-choice option. Although it did not reach a statistically significant level in pairwise comparison, the frequency of “void” in the two-choice option was greater in French than in Chinese and Japanese participants. For the two-choice question, the “void” response was not explicitly offered in the questionnaire, and Chinese and Japanese participants “wrote in” this response less often than the French participants, perhaps because the Chinese and Japanese are more collectivist and group-oriented (Nisbett et al., 2001), giving them a tendency not to depart from the given framework.

The present experiment uncovered cultural differences in conditional reasoning, but it is unclear what factors may affect such cultural differences, and our results have some implications for further research. Recent studies of conditionals (Skovgaard-Olsen et al., 2016; Vidal and Baratgin, 2017) have found evidence that people’s evaluations of conditionals, where there is no relevant link between the antecedent and consequent or antecedent is negatively associated with consequent, can violate the Equation *P*(*if p then q*) = *P*(*q|p*). In Westerners, the violation of the Equation is found for conditionals where *p* and *q* are independent or negatively associated. Our results here suggest that Easterners are more sensitive to contextual information such as relevance. It is possible then that there are cultural differences in perceived relevance, and that Easterners are more strongly affected by relevance and more frequently violate the Equation. There are also possible cultural differences in the interpretation of the “void” value. A meta-analytic review of truth-table results for conditionals (Schroyens, 2010) found that “irrelevant” judgments of false antecedent cases was a minor response for specific tasks (e.g., the implicit negation task), and “irrelevant” judgments actually meant neither true nor false and not irrelevant. The present experiment revealed that false antecedent cases were “void,” neither true/wins nor false/loses, cases for both Easterners and Westerners, but there were cultural differences in whether people considered void cases as relevant for the probability truth/winning or falsity/losing of IC/CB. Future research on three-valued responses will have to explore the detailed cultural differences in the meaning of the “void” value. Finally, individual differences in understanding conditionals will have to be investigated. The present study did not measure cognitive styles, but studies in cultural psychology have revealed that cultural differences in cognitive styles were stable (e.g., Choi et al., 2007; Na et al., 2010). Previous work attributed individual differences in conditional reasoning to individual differences in cognitive ability (e.g., Evans et al., 2007), but the present study suggests that individual differences in analytic vs. holistic cognitive styles may affect individual differences in conditional reasoning.

## Conclusion

Our results revealed cultural differences in judgments about the natural language conditional IC and CB. Easterners had a greater tendency to take whole cases, including void cases, into account, holistically judging the probability of a conditional to be *P*(*if s then b*) = *P*(*s*) *^∗^ P*(*b|s*). We noted that this holistic response includes the conditional probability *P*(*b|s*), and so Easterners may understand the conditional as suppositional, as *b given s*. In spite of Easterners’ greater holistic tendencies in detail, there was a high-level of similarity between their responses and those of Westerners, as predicted by Bayesian accounts of the natural language IC. The overall modal response for the probability of the conditional was the conditional probability, and conditionals were generally given a *de Finetti* table. The new Bayesian account of conditionals should look further for cultural differences, and future research will have to explore detailed cultural differences in contextual and relevance effects, the meaning of the “void” value and individual cultural differences in people’s understanding of conditionals.

## Ethics Statement

Because this experiment was conducted anonymously and satisfied the ethical requirements of Department of Human Behavior Science, Osaka City University, the experiment was required no ethical review by ethical committee.

## Author Contributions

HN, JS, JB, DO, TT and HY: study conception and design, interpretation of data, and critical revision. HN, JS, JB, and HY: acquisition of data. HN and JS: analysis of data. HN: drafting of manuscript.

## Conflict of Interest Statement

The authors declare that the research was conducted in the absence of any commercial or financial relationships that could be construed as a potential conflict of interest.

## Footnotes

1 French participants in Politzer et al.’s (2010) study completed high school and 0–10 years in higher education (average was 4 years). Both Chinese and Japanese students have fewer years of higher education than the French. But in China and Japan, there are selection competitions to enter universities, which is not the case in France. Overall, we can consider that these populations are comparable.
